# Structures of the reaction products of the AZADO radical with TCNQF_4_ or thiourea

**DOI:** 10.3762/bjoc.9.169

**Published:** 2013-07-25

**Authors:** Hideto Suzuki, Yuta Kawahara, Hiroki Akutsu, Jun-ichi Yamada, Shin’ichi Nakatsuji

**Affiliations:** 1 Graduate School of Material Science, University of Hyogo, 3-2-1 Kouto, Kamigori, Hyogo 678-1297, Japan, Tel: +81-791-58-0163; fax: +81-791-58-0164

**Keywords:** adduct, inclusion compound, nitroxide radical, TCNQF_4_, thiourea

## Abstract

While an addition product was formed by the reaction of AZADO (2-azaadamantane *N*-oxyl) with TCNQF_4_ (2,3,5,6-tetrafluoro-7,7,8,8-tetracyanoquinodimethane), the reaction of AZADO with thiourea provided an inclusion compound, in which AZADO molecules are incorporated in cylindrical channels formed by thiourea molecules.

## Introduction

TEMPO radical (2,2,6,6-tetramethylpiperidinyl-*N*-oxyl) (**1**) is a typical nitroxide radical and is persistent because of the steric hindrance of the four neighboring methyl groups of the NO moiety protecting it from attack by various reagents including oxygen [1]. Sometimes, however, the merit turns out to be a drawback by limiting its properties and reactivity. For example, **1** is inefficient in the oxidation of sterically hindered secondary alcohols.

The AZADO radical (**2**) [2] is an intriguing nitroxide radical with adamantane-like structure and less steric hindrance than the TEMPO radical (**1**). Radical **2** has recently been reported to be superior to the catalytic efficiency of **2** for oxidation of various alcohols [3]. Furthermore, it has been proved to display unique thermochromism and magnetic phase transitions [4]. More recently, it has been clarified to play a significant role as an efficient mediator for dye-sensitized solar cells with conversion efficiency as high as 8.6% [5].

**Figure 1:**
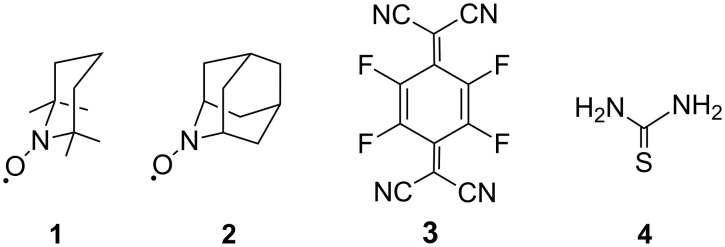
Structural formula of TEMPO (**1**), AZADO (**2**), TCNQF_4_ (**3**) and thiourea (**4**).

Previously, we observed that the TEMPO radical (**1**) forms corresponding charge transfer (CT) complexes with appropriate acceptors such as TCNQF_4_ (**3**) [6], and even more impressively some TEMPO derivatives with appropriate acceptor units such as 1,4-benzoquinone [7] or naphthalenediimide [8] can form single-component CT complexes by self-assembly. Based on previous results, we next intended to see if similar CT complexes would be formed from AZADO (**2**) with an appropriate acceptor. Moreover, we tried to prepare a supramolecule from **2** with thiourea (**4**), as structurally similar 1-bromoadamantane was reported to form an intriguing inclusion compound with thiouea [9]. There are some reported examples of inclusion compounds derived from TEMPO and related nitroxide radicals with host molecules such as tris(*o*-phenylenedioxy)cyclotriphosphazene (TPP) [10], cyclodextrin (CD) [11], or cucurbituril (CB) derivatives [12] but the inclusion compound derived from AZADO is still unknown. Here in this paper we wish to report the apparent difference of reactivity between AZADO (**2**) and TEMPO (**1**) with TCNQF_4_ (**3**) or thiourea (**4**) together with the structural motifs of the products derived from **2** (Figure 1).

## Results and Discussion

### Structure of the reaction product of AZADO and TCNQF_4_

**Scheme 1:**
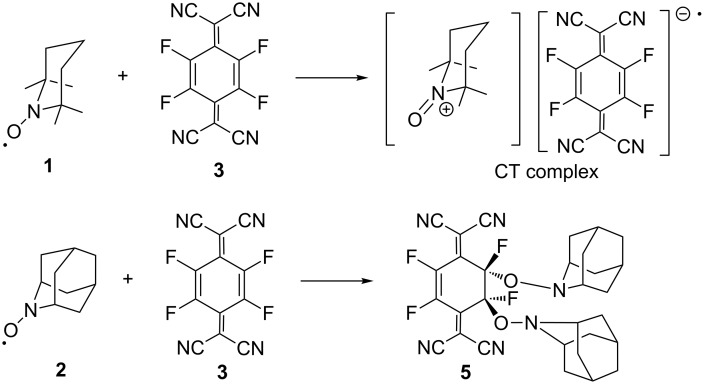
Difference of the reaction products from TEMPO (1) and AZADO (**2**) with TCNQF_4_ (**3**).

**Figure 2:**
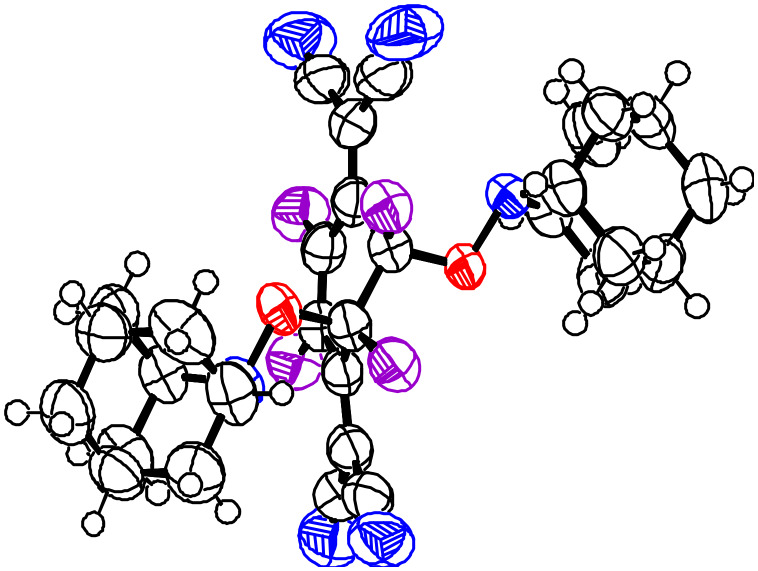
Molecular structure of the adduct **5** obtained by X-ray analysis.

When TEMPO (**1**) was reacted with an equimolecular amount of TCNQF_4_ (**3**) in acetonitrile, it was observed that the colour of the reaction mixture turned to deep green. The resulting dark blue crystals obtained after workup were found to be the CT complex with a radical-to-acceptor ratio of 1:1 being elucidated by its X-ray analysis [6]. On the other hand, the reaction of AZADO (**2**) with an equimolecular amount of TCNQF_4_ (**3**) did not give dark blue crystals but gave an orange solid. After purification, the orange crystals obtained were proved to be the addition product **5** and not a CT complex as seen from the X-ray analysis. Furthermore, the addition was found to take place to a double bond of the six-membered ring in a *trans*-manner, as seen from its molecular structure (Scheme 1) (Figure 2).

**Scheme 2:**
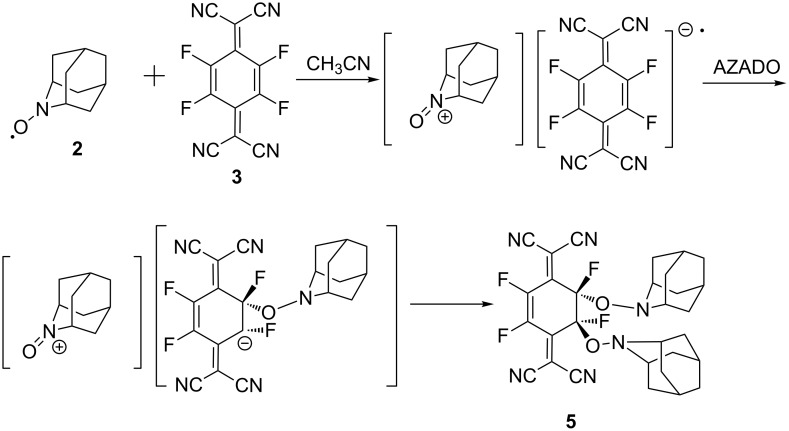
A plausible mechanism of the reaction of AZADO (**2**) with TCNQF_4_ (**3**).

Therefore, a plausible mechanism of the reaction could be considered as shown in Scheme 2. First, a transient CT complex would be formed between **2** and **3** but it would be unstable enough to be attacked by another AZADO molecule in the next step to give an addition product (a salt) resulting from the addition to one of the double bonds in the six-membered ring. Finally, the oxoammonium cation in the adduct will react with the counter anion from the less hindered site to afford the final product **5** with *trans*-configuration.

The cyclic voltammetry (CV) data of TEMPO (**1**), AZADO (**2**), TCNQF_4_ (**3**) and the adduct **5** are summarized in Table 1. The lower first oxidation potential (*E*_1_^OX^) of AZADO than that of TEMPO suggests that the reaction between AZADO and TCNQF_4_ can take place slightly more easily than that between TEMPO and TCNQF_4_. The comparison of the reduction potential of **3** and **5** indicates that the first and the second reduction potentials of **5** apparently decrease compared to those of **3**, and the first reduction potential of **5** (0.02 V) just corresponds to the second reduction potential of **3** by chance (Table 1). Nevertheless, it still has a weak acceptor ability as anticipated from the values.

**Table 1:** CV (cyclic voltammetry) data of TEMPO (**1**), AZADO (**2**), TCNQF_4_ (**3**) and adduct **5**.^a^

Compound	*E*_1_^OX^	*E*_1_^RED^	*E*_2_^RED^

**1**	0.80	–	–
**2**	0.77	–	–
**3**	–	0.55	0.02
**5**	–	0.02	−0.42

^a^V versus SCE, 0.1 M *n*-Bu_4_NClO_4_ in dichloromethane. Scan rate 50 mV/s at rt.

**Figure 3:**
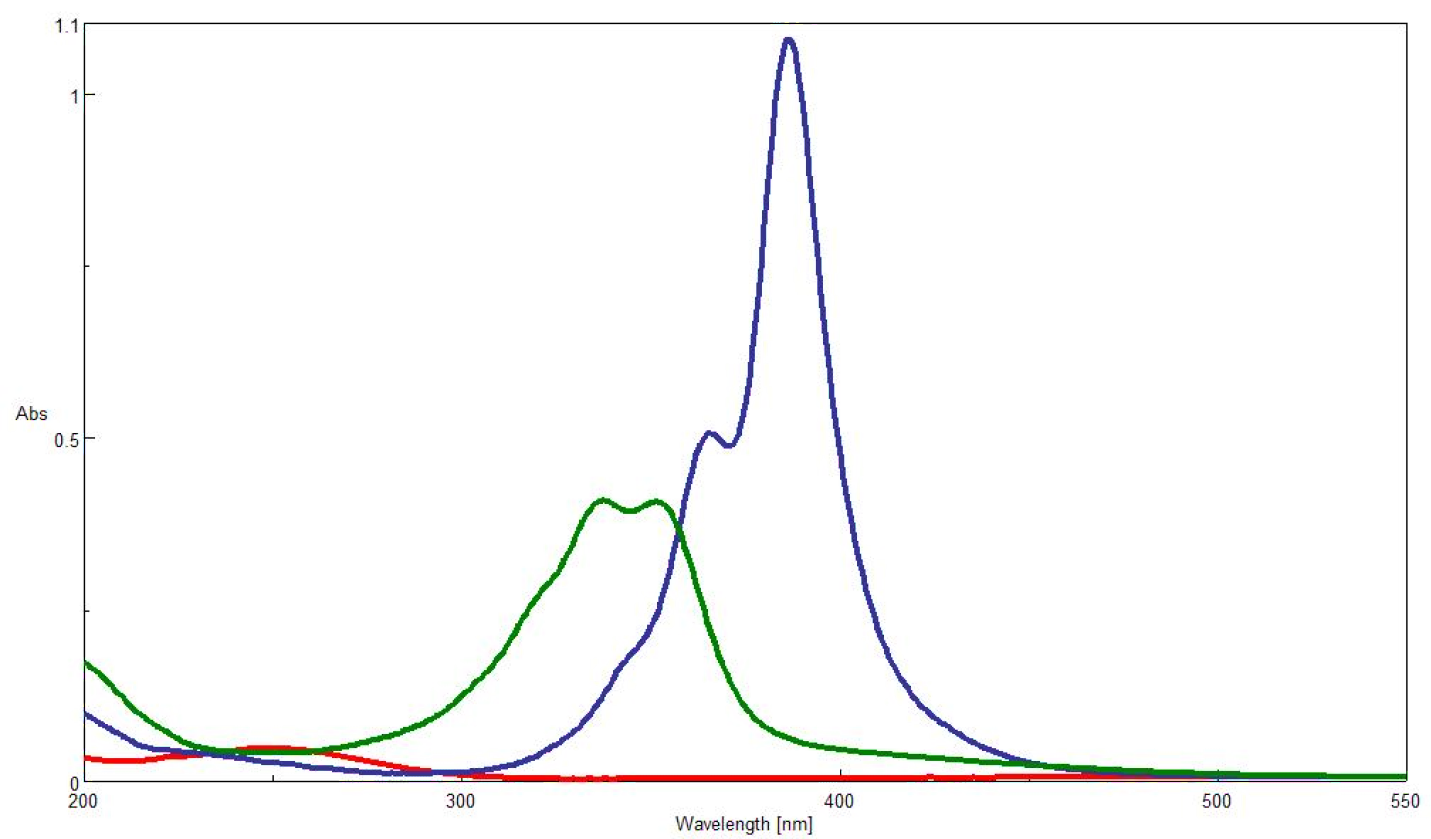
UV–vis spectra of **2** (red line), **3** (blue line) and **5** (green line).

The UV–vis spectra of AZADO (**2**), TCNQF_4_ (**3**) and the adduct **5** in acetonitrile solution are shown in Figure 3. Only very weak and broad absorptions could be discriminated for AZADO radical (**2**) at around 250 nm and at 450 nm and the latter one is due to a forbidden transition. Whereas sharp absorptions are observed for TCNQF_4_ (**3**) at around 365 nm (shoulder) and 390 nm, those of adduct **5** are broad and appear in a shorter wavelength region, reflecting apparently the conversion of a C–C double bond to a single one.

### Structure of the reaction product of AZADO and thiourea

**Scheme 3:**
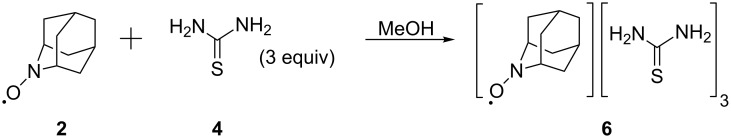
The reaction of **2** with **4** to form **6**.

**Figure 4:**
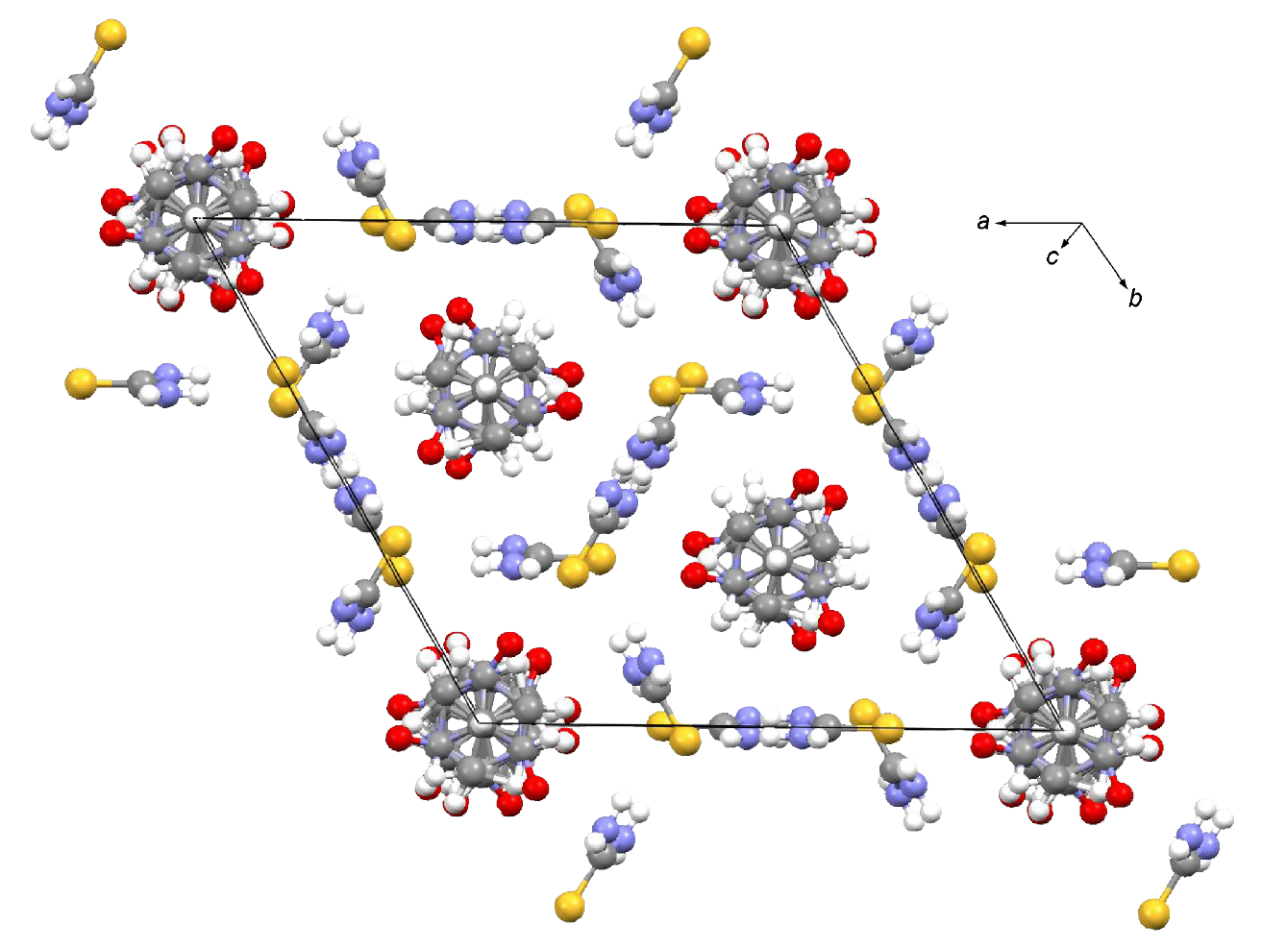
Crystal structure of the inclusion compound **6** obtained by X-ray analysis.

We next tried to perform the reaction of AZADO (**2**) with thiourea (**4**) to see if some intriguing complexes would be formed, since a lot of inclusion compounds derived from urea or thiourea are known [13]. The reaction between **2** and **4** was first tried by mixing each component in acetonitrile solution but it was unsuccessful in obtaining a relevant solid. However, a brownish solid could be obtained when a similar experiment was carried out in methanol solution and that was proved after purification to be an inclusion compound as elucidated by X-ray analysis (Scheme 3) (Figure 4).

As shown in Figure 4, the molecules of AZADO (**2**) are incorporated in cylindrical channels formed by thiourea molecules (**4**) along the *c*-axis and the ratio of **2** and **4** was proved to be 1:3 in this case as elucidated by elemental analysis, while that of bromoadamantane and **4** was reported to be 1:4 [9]. As usually seen in thiourea-derived inclusion compounds, the thiourea channels are formed by hydrogen bonds between the amino-hydrogen atoms and thiocarbonyl groups of neighbouring thiourea molecules to interlock honeycomb-type structures. There exist apparent short contacts of 3.06(5) Å between the oxygen atoms of the guest molecules and the sulfur atoms of host molecules and the interactions derived from the short contacts are supposed to be responsible at least in part for retaining the guest molecules inside the channels together with other weak nonbonding interactions as usually seen in other thiourea-derived complexes [13]. It is anticipated from the X-ray data at room temperature that AZADO molecules within the thiourea channels are disordered and it appears to be due to dynamic interconversion between the six orientations with essentially equal occupancies of the radical molecules within the channels [9], although the single crystal data at low temperatures are not available yet and the distinct feature of the disorder is not clarified at present.

Quite similar λ_max_-values of the inclusion compound **6** are found to those of thiourea (**4**) in their UV–vis absorption spectra (see Supporting Information File 1) and that suggests the absence of any CT interaction between the guest **2** and the host molecules **4** when the inclusion compound **6** is dissolved and dissociated in the solvent.

On the other hand, TEMPO radical (**1**) did not form any inclusion compound under the same conditions with thiourea (**4**), but each component was recovered. This was a rather unexpected result, because some precedent examples of TEMPO-based inclusion complexes are actually known [10–12], but this is supposed to be due to the steric hindrance of the extra methyl substituents of the TEMPO radical, and thus, a sharp difference was clear between AZADO and TEMPO in the formation of an inclusion compound.

## Conclusion

AZADO radical (**2**) was found to react with TCNQF_4_ (**3**) to provide an addition product **5** with *trans*-configuration and to form an inclusion compound **6** with thiourea (**4**), thus showing a marked difference in reactivity compared with that of TEMPO radical (**1**), due at least in part to the difference of the steric effect of the extra methyl substituents of the latter radical.

## Supporting Information

File 1 Experimental procedures and summary of crystal data of **5** and **6**.
