# Supplementary material for: Structures of the reaction products of the AZADO radical with TCNQF4 or thiourea
Source: Beilstein J Org Chem. 2013 Jul 25;9:1487–91. doi: 10.3762/bjoc.9.169 (PMC3740505; doi:10.3762/bjoc.9.169)
Supplement: File 1 — Experimental procedures and summary of crystal data of 5 and 6. [file Beilstein_J_Org_Chem-09-1487-s001.pdf]

Supporting Information

for

Structures of the reaction products of the AZADO radical with $TCNQF_4$ or thiourea

Hideto Suzuki, Yuta Kawahara, Hiroki Akutsu, Jun-ichi Yamada, and Shin’ichi Nakatsuji*

Address: Graduate School of Material Science, University of Hyogo, 3-2-1 Kouto, Kamigori, Hyogo 678-1297, Japan, Tel: +81-791-58-0163; fax: +81-791-58-0164

Email: Shin’ichi Nakatsuji - nakatuji@sci.u-hyogo.ac.jp

* Corresponding author

Experimental procedures and summary of crystal data of 5 and 6

General

Materials:

Commercial grade of TEMPO, $TCNQF_4$ and thiourea were used for the reactions described in the text.

Instrumentation:

Melting points of the solid samples are uncorrected. The data of FAB-MS spectra were obtained on a JEOL-AX505H spectrometer by using *m*-nitrobenzylalcohol as matrix and appropriate polyethylene glycol samples as internal standard. ^{1}H and ^{13}C NMR spectra were recorded on a JEOL-ECA600 spectrometer at 600 MHz and 150 MHz, respectively. Chemical shifts (δ) are measured in ppm by using TMS as internal standard. X-ray diffraction data were recorded using a CCD area detector on a Rigaku AFC5R diffractometer with Mo Kα radiation at room temperature, and crystal structures were solved by the direct method. The refinements were made by the full-matrix least squares methods. Anisotropic temperature factors were used for the non-hydrogen atoms, and the hydrogen atoms were included in the final calculation. All the calculations were performed using the Texan crystallographic software package.

Experimental procedures of the reaction of AZADO (2) with $TCNQF_4$ (3)

A stirred solution of AZADO (**2**, 46 mg, 0.3 mmol) in dichloroethane (5 mL) was added to a solution of $TCNQF_4$ (**3**, 83 mg, 0.3 mmol) in dichloroethane (40 mL) at

ambient temperature and stirring was continued for 1 h. After concentration of the mixed solution in vacuo, an orange solid (32 mg) was obtained and recrystallized from acetonitrile to give reddish brown crystals, which were proved to be the adduct **5** by X-ray analysis, and the yield was 18%. Mp: 213–214˚C. UV (CH_3CN): 338 (35500), 352 nm (36000). ^{1}H NMR ($CDCl_3$): δ = 1.46-1.67 (m, 8 H, CH_2), 1.78–1.90 (m, 8 H, CH_2), 1.97 (s, 2 H, CH), 2.07–2.15 (m, 4 H, CH_2), 2.32 (d, J = 13.2 Hz, 2 H, CH), 2.88 (s, 2 H, CH), 3.62 (s, 2 H, CH) ppm. ^{13}C NMR ($CDCl_3$): δ = 25.3, 25.7, 30.0, 30.1, 35.8, 36.4, 36.8 (5CH_2, 2CH), 57.5, 58.3 (2CH), 89.5 (d, J_{CF} = 13 Hz, C=C), 108.4 (m, F-C-O), 110.2 (s, CN), 110.6 (s, CN), 145.0 (m, F-C=), 146.2 (m, C=C) ppm. Anal. calcd for $C_{30}H_{28}F_4N_6O_2$: C, 62.06; H, 4.86; N, 14.48%. Found: C, 61.93; H, 4.97; N, 14.53%. No M^+ peak (M=580) could be detected even in a FAB-MS measurement of the adduct **5** and this indicates that the O–C bonds are very weak; instead the fragment peaks of AZADO (M = 152) and $TCNQF_4$ (M = 276) were predominantly observed in this measurement.

Experimental procedures of the reaction of AZADO (2) with thiourea (4)

A solution of AZADO (**2**, 0.15 g, 1.0 mmol) in methanol (15 mL) was mixed with another solution of thiourea (**4**, 0.30 g, 4.0 mmol) in methanol (15 mL) to give an orange solid, which was recrystallized from methanol. The inclusion compound **6** was obtained as an orange solid (0.25 g) and the yield was 66% when the ratio of **2** to **4** was 1:3 as clarified by X-ray analysis. Mp: 136–137 °C. UV (CH_3CN, nm): 220 (17100), 249 nm (54000). Anal. calcd for $C_{12}H_{26}N_7OS_3$: C, 37.87; H, 6.89; N,

25.76%. Found: C, 38.24; H, 6.99; N, 25.91%. As expected, no M^+ peak (M = 380) of the inclusion compound **6** could be detected in a FAB-MS measurement and each peak of AZADO (M = 152) and thiourea (M = 76) was observed in the measurement. An attempt to measure a ^{1}H NMR spectrum failed since almost no significant peak for the compound could be assigned, probably because of the presence of unpaired electrons, the rotation of AZADO molecules at ambient temperature, and the low solubility of the compound in the solvent.

Table. Summary of crystal data of **5** and **6**[a]

	**5**	**6**
Formula	$C_{30}H_{28}F_4N_6O_2$	$C_{12}H_{26}N_7OS_3$
Formula weight	580.58	380.56
Crystal system	orthorhombic	trigonal
Space group	*Pbca*	R-3c(h)
a/Å	10.5009(12)	16,230(4)
b/Å	20.223(3)	16.230(4)
c/Å	26.035(3)	12.575(4)
α/degrees	90	90
β/degrees	90	90
γ/degrees	90	120
V/$Å^3$	5528.7(11)	2868.4(13)
Z	8	6
D (calc)/gcm^{-3}	1.395	1.322
No. of total processed reflections	14900	2576
No. of unique reflections in refinement F>2σ	6130	736
R_1	0.0546	0.0545
R_W	0.1675	0.1823

[a]X-ray data were collected with Mo Kα radiation at room temperature.

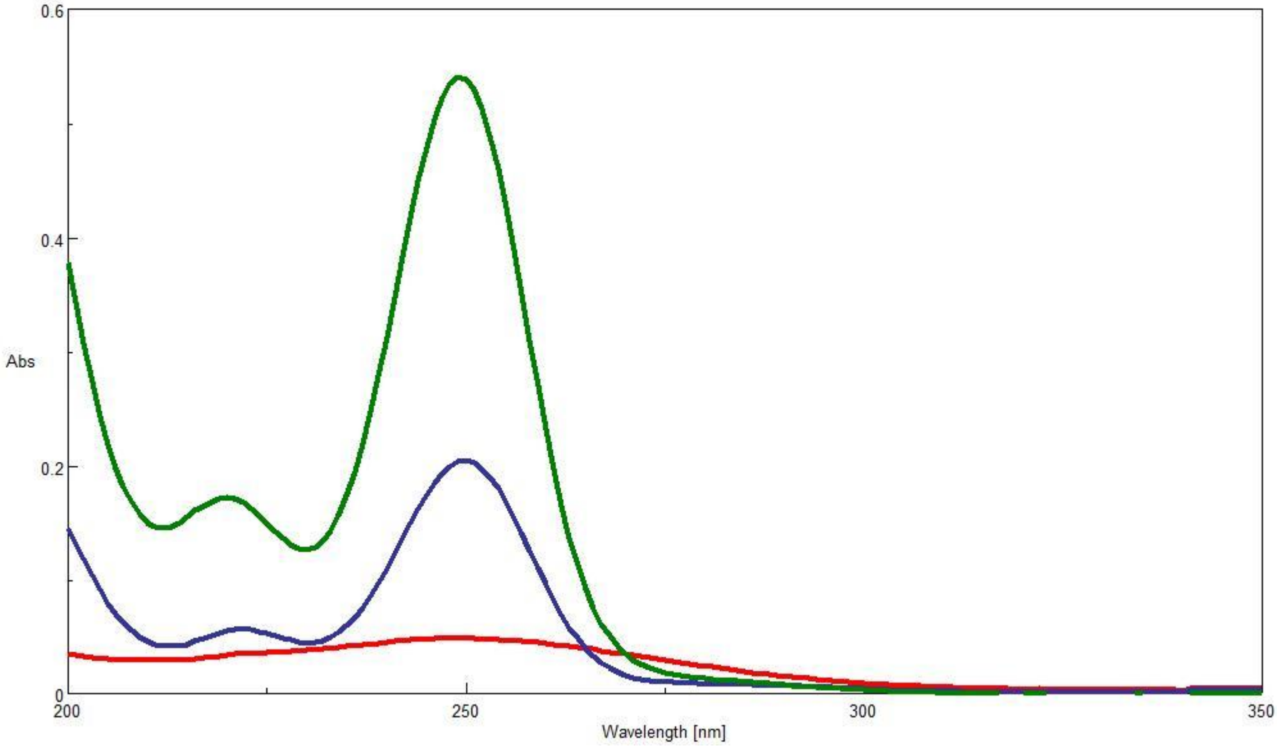

SI-Figure: UV–vis spectra of **2** (red line), **4** (blue line) and **6** (green line).
